# Tris(3-chloro­pentane-2,4-dionato-κ^2^ *O*,*O*′)iron(III)

**DOI:** 10.1107/S1600536812023215

**Published:** 2012-05-26

**Authors:** Franc Perdih

**Affiliations:** a Faculty of Chemistry and Chemical Technology, University of Ljubljana, Aškerčeva 5, PO Box 537, SI-1000 Ljubljana, Slovenia; b CO EN–FIST, Dunajska 156, SI-1000 Ljubljana, Slovenia

## Abstract

In the title compound, [Fe(C_5_H_6_ClO_2_)_3_], the Fe^III^ cation is situated on a twofold rotation axis and is coordinated by six O atoms from three 3-chloro­pentane-2,4-dionate ligands in a slightly distorted octa­hedral environment. Fe—O bond lengths are in the range 1.9818 (18)–1.9957 (18) Å. The *trans* O—Fe—O angles are 169.06 (13) and 171.54 (8)°, whereas the corresponding *cis* angles are in the range 84.81 (10)–100.68 (12)°. In the crystal, mol­ecules are linked *via* C—H⋯Cl inter­actions.

## Related literature

For applications of metal complexes with β-diketonate ligands, see: Bray *et al.* (2007 ▶); Garibay *et al.* (2009 ▶); Perdih (2011 ▶); Schröder *et al.* (2011 ▶). For related structures, see: Iball & Morgan (1967 ▶); Perdih (2012 ▶); Pfluger & Haradem (1983 ▶).
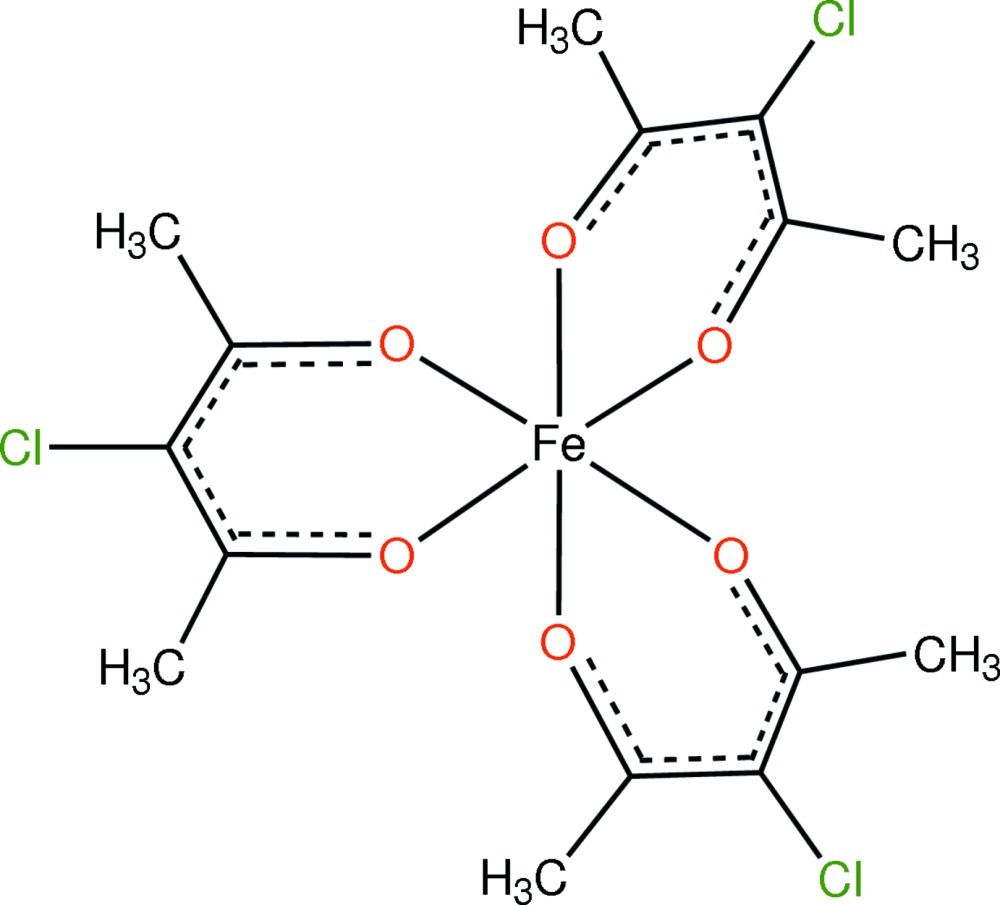


## Experimental

### 

#### Crystal data

[Fe(C_5_H_6_ClO_2_)_3_]
*M* *_r_* = 456.49
Monoclinic, 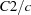

*a* = 15.7745 (4) Å
*b* = 9.5424 (2) Å
*c* = 12.9833 (3) Å
β = 100.610 (1)°
*V* = 1920.92 (8) Å^3^
*Z* = 4
Mo *K*α radiation
μ = 1.23 mm^−1^
*T* = 293 K
0.25 × 0.25 × 0.13 mm

#### Data collection

Nonius KappaCCD area-detector diffractometer
Absorption correction: multi-scan (*SCALEPACK*; Otwinowski & Minor, 1997 ▶) *T*_min_ = 0.749, *T*_max_ = 0.857
4155 measured reflections
2155 independent reflections
1927 reflections with *I* > 2σ(*I*)
*R*_int_ = 0.012

#### Refinement

*R*[*F* ^2^ > 2σ(*F* ^2^)] = 0.044
*wR*(*F* ^2^) = 0.127
*S* = 1.07
2155 reflections
118 parameters
H-atom parameters constrained
Δρ_max_ = 0.88 e Å^−3^
Δρ_min_ = −0.62 e Å^−3^

### 

Data collection: *COLLECT* (Hooft, 1998 ▶); cell refinement: *DENZO-SMN* (Otwinowski & Minor, 1997 ▶); data reduction: *DENZO-SMN*; program(s) used to solve structure: *SHELXS97* (Sheldrick, 2008 ▶); program(s) used to refine structure: *SHELXL97* (Sheldrick, 2008 ▶); molecular graphics: *ORTEP-3 for Windows* (Farrugia, 1997 ▶) and *DIAMOND* (Brandenburg, 1999 ▶); software used to prepare material for publication: *WinGX* (Farrugia, 1999 ▶) and *publCIF* (Westrip, 2010 ▶).

## Supplementary Material

Crystal structure: contains datablock(s) I, global. DOI: 10.1107/S1600536812023215/im2375sup1.cif

Structure factors: contains datablock(s) I. DOI: 10.1107/S1600536812023215/im2375Isup2.hkl

Additional supplementary materials:  crystallographic information; 3D view; checkCIF report

## Figures and Tables

**Table 1:** Hydrogen-bond geometry (Å, °)

*D*—H⋯*A*	*D*—H	H⋯*A*	*D*⋯*A*	*D*—H⋯*A*
C6—H6*A*⋯Cl1^i^	0.96	2.78	3.642 (3)	150

Symmetry code: (i) 
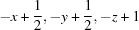
.

**Fig. 1.**
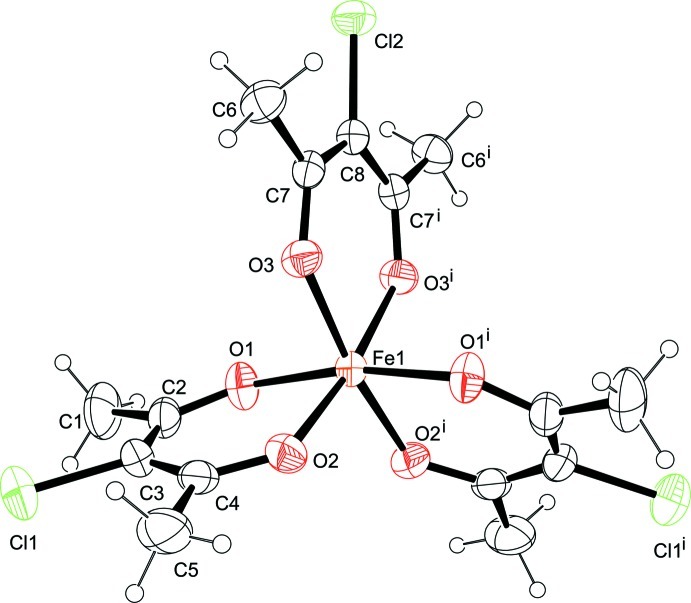
Molecular structure of the title complex showing displacement ellipsoids at the 30% probability level. Symmetry code: i = –x + 1, y, –z + 3/2.

**Fig. 2.**
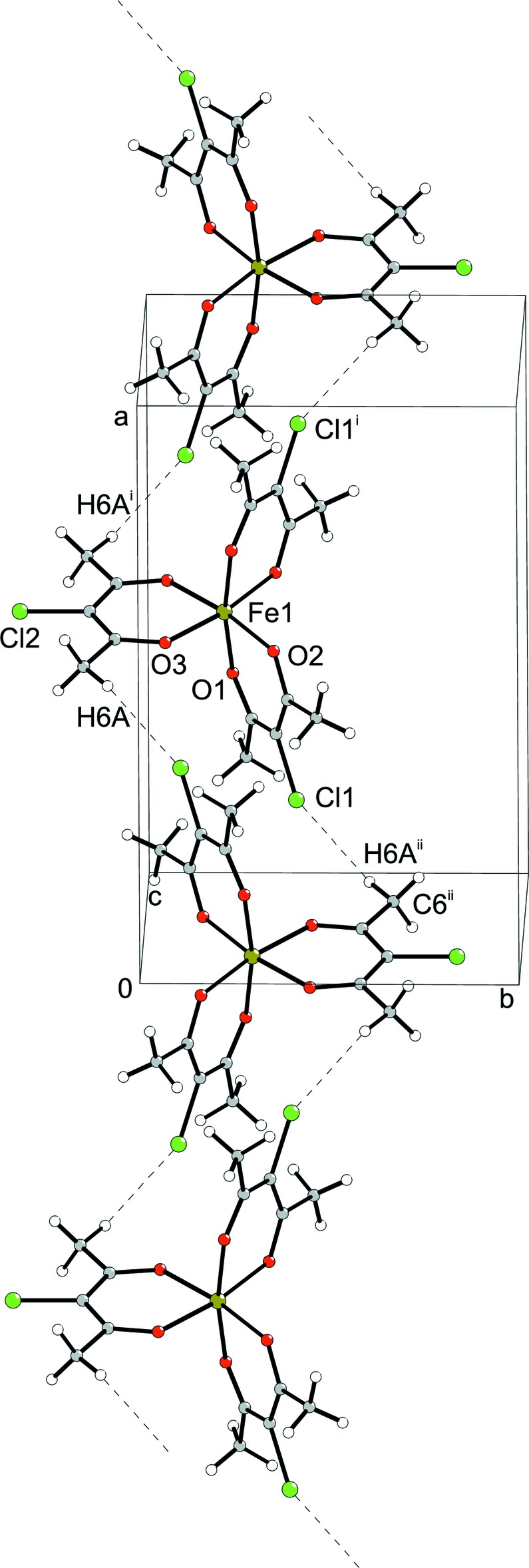
1D infinte chain with dashed lines indicating intermolecular C6—H6A···Cl1 hydrogen bonding. For the sake of clarity, H atoms not involved in the motif shown have been omitted. Symmetry code: ii = –x + 1/2, –y + 1/2, –z + 1.

## supplementary crystallographic information

### Comment

β-Diketonates have been proven to be versatile ligands for various metal ions. They can be easily derivatized, thus modifying the electronic and steric nature of these ligands to design suitable structure/function relationships (Bray *et al.*, 2007; Garibay *et al.*, 2009; Perdih (2011). Metal-organic frameworks are considered as promising materials for many applications mostly due to interesting porosity properties. Besides the potential applications as gas storage other applications such as molecular sensing, ion exchange, catalysis, optics and magnetism have received considerable attention (Bray *et al.*, 2007; Garibay *et al.*, 2009). Particularly interesting is the metal-ligand coordination with applications in organic synthesis, where iron β-diketonate compounds showed great applicability. Reasons for this are the natural abundance of this metal and also it's biocompatibility, both of which are essential for the development of sustainable chemical catalysis (Schröder *et al.*, 2011).

In the title molecule (Fig. 1), the iron(III) cation is situated on a twofold axis, and is surrounded by six O atoms from three 3-chloropentane-2,4-dionate ligands in a slightly distorted octahedral environment. Fe—O bond lengths are in the range of 1.9818 (18)–1.9957 (18) Å, *trans* O—Fe—O angles are 169.06 (13)° and 171.54 (8)°, and *cis* angles are in the range of 84.81 (10)°–100.68 (12)°. These bond lengths are similar as for example in Fe(acac)_3_ (Iball & Morgan, 1967). The title compound is isostructural with the corresponding aluminium(III) compound (Perdih, 2012). The displacement of the metal atom is best described by a bending of a chelate ligand about the "bite" atoms. The angles between the O—Fe—O and the ligand chelate mean planes are 0.78° and 12.68°. For comparison these values are 1.40°, 10.13° and 11.98° in Fe(hfac)_3_ (hfac = hexafluoroacetylacetonate) (Pfluger & Haradem, 1983) and 0.05°, 3.24° and 10.60° in Fe(acac)_3_ (Ibell & Morgan, 1967). A 1-D framework is achieved due to weak intermolecular C6–H6A···Cl1 (–*x* + 1/2, –*y* + 1/2, –*z* + 1) interactions where one 3-chloropentane-2,4-dionate ligand acts as a hydrogen-bond donors and two ligands are hydrogen-bond acceptors (Fig. 2).

### Experimental

To a clear solution of FeCl_3_^.^ H_2_O (2 mmol, 0.54 g) in water (15 ml) a solution of 3-chloropentane-2,4-dione (6 mmol, 0.81 g) in methanol (5 ml) was added while stirring. Afterwards 1 *M* NaOH (6 ml) was slowly added and the resulting solution was stirred at 70°C for 15 minutes. After cooling to room temperature the deep red product was filtrated, washed with water (20 ml), and subsequently air-dried. Yield: 0.65 g, 71%. Crystals suitable for X-ray analysis were obtained by recrystallization from ethanol.

### Refinement

All H atoms were initially located in a difference Fourier maps and were subsequently treated as riding atoms in geometrically idealized positions, with C—H = 0.96 Å, and with *U*_iso_(H) = 1.5*U*_eq_(C). To improve the refinement results, two reflections with too high value of *δ*(*F*^2^)/e.s.d. and with *F*_o_^2^ < *F*_c_^2^ were deleted from the refinement.

### Crystal data

**Table:**

[Fe(C_5_H_6_ClO_2_)_3_]	*F*(000) = 932
*M**_r_* = 456.49	*D*_x_ = 1.578 Mg m^−^^3^
Monoclinic, *C*2/*c*	Mo *K*α radiation, λ = 0.71073 Å
Hall symbol: -C 2yc	Cell parameters from 2278 reflections
*a* = 15.7745 (4) Å	θ = 2.6–27.5°
*b* = 9.5424 (2) Å	µ = 1.23 mm^−^^1^
*c* = 12.9833 (3) Å	*T* = 293 K
β = 100.610 (1)°	Prism, red
*V* = 1920.92 (8) Å^3^	0.25 × 0.25 × 0.13 mm
*Z* = 4	

### Data collection

**Table:**

Nonius KappaCCD area-detector diffractometer	2155 independent reflections
Radiation source: fine-focus sealed tube	1927 reflections with *I* > 2σ(*I*)
Graphite monochromator	*R*_int_ = 0.012
Detector resolution: 0.055 pixels mm^-1^	θ_max_ = 27.4°, θ_min_ = 3.9°
ω scans	*h* = −20→20
Absorption correction: multi-scan (*SCALEPACK*; Otwinowski & Minor, 1997)	*k* = −12→12
*T*_min_ = 0.749, *T*_max_ = 0.857	*l* = −16→16
4155 measured reflections	

### Refinement

**Table:**

Refinement on *F*^2^	Primary atom site location: structure-invariant direct methods
Least-squares matrix: full	Secondary atom site location: difference Fourier map
*R*[*F*^2^ > 2σ(*F*^2^)] = 0.044	Hydrogen site location: inferred from neighbouring sites
*wR*(*F*^2^) = 0.127	H-atom parameters constrained
*S* = 1.07	*w* = 1/[σ^2^(*F*_o_^2^) + (0.0748*P*)^2^ + 1.6605*P*] where *P* = (*F*_o_^2^ + 2*F*_c_^2^)/3
2155 reflections	(Δ/σ)_max_ < 0.001
118 parameters	Δρ_max_ = 0.88 e Å^−^^3^
0 restraints	Δρ_min_ = −0.62 e Å^−^^3^

### Special details

**Table:**

Experimental. 192 frames in 5 sets of ω scans. Rotation/frame = 2.0 °. Crystal-detector distance = 25.00 mm. Measuring time = 60 s/°.
Geometry. All s.u.'s (except the s.u. in the dihedral angle between two l.s. planes) are estimated using the full covariance matrix. The cell s.u.'s are taken into account individually in the estimation of s.u.'s in distances, angles and torsion angles; correlations between s.u.'s in cell parameters are only used when they are defined by crystal symmetry. An approximate (isotropic) treatment of cell s.u.'s is used for estimating s.u.'s involving l.s. planes.
Refinement. Refinement of *F*^2^ against ALL reflections. The weighted *R*-factor *wR* and goodness of fit *S* are based on *F*^2^, conventional *R*-factors *R* are based on *F*, with *F* set to zero for negative *F*^2^. The threshold expression of *F*^2^ > 2*σ*(*F*^2^) is used only for calculating *R*-factors(gt) *etc*. and is not relevant to the choice of reflections for refinement. *R*-factors based on *F*^2^ are statistically about twice as large as those based on *F*, and *R*-factors based on ALL data will be even larger.

### Fractional atomic coordinates and isotropic or equivalent isotropic displacement parameters (Å^2^)

**Table:**

	*x*	*y*	*z*	*U*_iso_*/*U*_eq_	
Fe1	0.5	0.20825 (5)	0.75	0.04488 (18)	
Cl1	0.20617 (6)	0.40123 (14)	0.58549 (12)	0.1198 (5)	
Cl2	0.5	−0.33055 (10)	0.75	0.0684 (3)	
O1	0.38591 (12)	0.2280 (2)	0.79173 (16)	0.0642 (5)	
O2	0.45438 (12)	0.3417 (2)	0.63475 (15)	0.0593 (4)	
O3	0.46568 (12)	0.05417 (17)	0.64737 (13)	0.0528 (4)	
C1	0.2404 (2)	0.2638 (6)	0.7991 (4)	0.0987 (13)	
H1A	0.261	0.2436	0.8718	0.148*	
H1B	0.2042	0.1885	0.7677	0.148*	
H1C	0.2077	0.3493	0.7928	0.148*	
C2	0.31593 (17)	0.2797 (3)	0.7439 (3)	0.0622 (7)	
C3	0.30871 (17)	0.3467 (3)	0.6477 (3)	0.0680 (8)	
C4	0.37767 (19)	0.3776 (3)	0.5971 (2)	0.0613 (7)	
C5	0.3663 (3)	0.4565 (4)	0.4949 (3)	0.0931 (12)	
H5A	0.4217	0.4738	0.477	0.14*	
H5B	0.338	0.5441	0.502	0.14*	
H5C	0.3318	0.4018	0.4407	0.14*	
C6	0.44210 (19)	−0.1585 (3)	0.5553 (2)	0.0621 (6)	
H6A	0.4189	−0.0946	0.5001	0.093*	
H6B	0.3986	−0.2251	0.5649	0.093*	
H6C	0.4906	−0.207	0.5371	0.093*	
C7	0.47040 (14)	−0.0786 (2)	0.65475 (17)	0.0450 (5)	
C8	0.5	−0.1469 (3)	0.75	0.0458 (6)	

### Atomic displacement parameters (Å^2^)

**Table:**

	*U*^11^	*U*^22^	*U*^33^	*U*^12^	*U*^13^	*U*^23^
Fe1	0.0379 (3)	0.0458 (3)	0.0498 (3)	0	0.00487 (18)	0
Cl1	0.0618 (5)	0.1015 (7)	0.1759 (12)	0.0164 (5)	−0.0313 (6)	0.0217 (8)
Cl2	0.0768 (6)	0.0450 (5)	0.0797 (6)	0	0.0049 (5)	0
O1	0.0414 (9)	0.0889 (14)	0.0627 (11)	0.0061 (9)	0.0104 (8)	0.0053 (10)
O2	0.0604 (10)	0.0518 (10)	0.0645 (10)	0.0054 (8)	0.0086 (8)	0.0092 (8)
O3	0.0610 (10)	0.0481 (9)	0.0457 (8)	−0.0038 (7)	0.0005 (7)	0.0015 (7)
C1	0.0466 (17)	0.135 (4)	0.119 (3)	0.0057 (19)	0.0246 (18)	−0.012 (3)
C2	0.0405 (12)	0.0661 (16)	0.0778 (17)	0.0025 (11)	0.0050 (11)	−0.0189 (13)
C3	0.0476 (13)	0.0521 (14)	0.095 (2)	0.0087 (11)	−0.0113 (13)	−0.0039 (14)
C4	0.0675 (16)	0.0383 (11)	0.0687 (15)	0.0023 (10)	−0.0117 (12)	−0.0008 (11)
C5	0.115 (3)	0.0650 (19)	0.085 (2)	−0.0019 (19)	−0.019 (2)	0.0217 (17)
C6	0.0720 (17)	0.0623 (15)	0.0495 (13)	−0.0082 (13)	0.0049 (11)	−0.0076 (11)
C7	0.0369 (10)	0.0515 (12)	0.0462 (11)	−0.0034 (8)	0.0066 (8)	−0.0030 (9)
C8	0.0390 (14)	0.0463 (16)	0.0520 (16)	0	0.0079 (12)	0

### Geometric parameters (Å, º)

**Table:**

Fe1—O1^i^	1.9818 (18)	C1—H1C	0.96
Fe1—O1	1.9818 (18)	C2—C3	1.388 (5)
Fe1—O3	1.9912 (17)	C3—C4	1.402 (4)
Fe1—O3^i^	1.9912 (17)	C4—C5	1.507 (4)
Fe1—O2^i^	1.9957 (18)	C5—H5A	0.96
Fe1—O2	1.9957 (18)	C5—H5B	0.96
Cl1—C3	1.749 (3)	C5—H5C	0.96
Cl2—C8	1.753 (3)	C6—C7	1.495 (3)
O1—C2	1.263 (3)	C6—H6A	0.96
O2—C4	1.266 (3)	C6—H6B	0.96
O3—C7	1.271 (3)	C6—H6C	0.96
C1—C2	1.507 (5)	C7—C8	1.400 (3)
C1—H1A	0.96	C8—C7^i^	1.400 (3)
C1—H1B	0.96		
			
O1^i^—Fe1—O1	169.06 (13)	C3—C2—C1	122.2 (3)
O1^i^—Fe1—O3	92.07 (8)	C2—C3—C4	125.2 (2)
O1—Fe1—O3	96.00 (9)	C2—C3—Cl1	117.9 (2)
O1^i^—Fe1—O3^i^	96.00 (9)	C4—C3—Cl1	116.9 (2)
O1—Fe1—O3^i^	92.07 (8)	O2—C4—C3	122.1 (3)
O3—Fe1—O3^i^	84.81 (10)	O2—C4—C5	115.2 (3)
O1^i^—Fe1—O2^i^	85.63 (8)	C3—C4—C5	122.7 (3)
O1—Fe1—O2^i^	87.39 (8)	C4—C5—H5A	109.5
O3—Fe1—O2^i^	171.54 (8)	C4—C5—H5B	109.5
O3^i^—Fe1—O2^i^	87.33 (8)	H5A—C5—H5B	109.5
O1^i^—Fe1—O2	87.39 (8)	C4—C5—H5C	109.5
O1—Fe1—O2	85.63 (8)	H5A—C5—H5C	109.5
O3—Fe1—O2	87.33 (8)	H5B—C5—H5C	109.5
O3^i^—Fe1—O2	171.54 (8)	C7—C6—H6A	109.5
O2^i^—Fe1—O2	100.68 (12)	C7—C6—H6B	109.5
C2—O1—Fe1	131.2 (2)	H6A—C6—H6B	109.5
C4—O2—Fe1	130.47 (19)	C7—C6—H6C	109.5
C7—O3—Fe1	132.86 (15)	H6A—C6—H6C	109.5
C2—C1—H1A	109.5	H6B—C6—H6C	109.5
C2—C1—H1B	109.5	O3—C7—C8	122.4 (2)
H1A—C1—H1B	109.5	O3—C7—C6	116.0 (2)
C2—C1—H1C	109.5	C8—C7—C6	121.6 (2)
H1A—C1—H1C	109.5	C7—C8—C7^i^	124.5 (3)
H1B—C1—H1C	109.5	C7—C8—Cl2	117.75 (16)
O1—C2—C3	122.8 (3)	C7^i^—C8—Cl2	117.75 (16)
O1—C2—C1	115.0 (3)		
			
O1^i^—Fe1—O1—C2	64.1 (3)	C1—C2—C3—C4	173.3 (3)
O3—Fe1—O1—C2	−73.3 (3)	O1—C2—C3—Cl1	175.1 (2)
O3^i^—Fe1—O1—C2	−158.3 (3)	C1—C2—C3—Cl1	−5.2 (4)
O2^i^—Fe1—O1—C2	114.5 (3)	Fe1—O2—C4—C3	13.5 (4)
O2—Fe1—O1—C2	13.6 (3)	Fe1—O2—C4—C5	−167.0 (2)
O1^i^—Fe1—O2—C4	170.7 (2)	C2—C3—C4—O2	2.2 (5)
O1—Fe1—O2—C4	−17.7 (2)	Cl1—C3—C4—O2	−179.3 (2)
O3—Fe1—O2—C4	78.5 (2)	C2—C3—C4—C5	−177.2 (3)
O2^i^—Fe1—O2—C4	−104.2 (2)	Cl1—C3—C4—C5	1.3 (4)
O1^i^—Fe1—O3—C7	93.7 (2)	Fe1—O3—C7—C8	4.1 (3)
O1—Fe1—O3—C7	−93.7 (2)	Fe1—O3—C7—C6	−176.07 (17)
O3^i^—Fe1—O3—C7	−2.14 (17)	O3—C7—C8—C7^i^	−2.01 (16)
O2—Fe1—O3—C7	−179.0 (2)	C6—C7—C8—C7^i^	178.2 (2)
Fe1—O1—C2—C3	−5.3 (4)	O3—C7—C8—Cl2	177.99 (16)
Fe1—O1—C2—C1	174.9 (2)	C6—C7—C8—Cl2	−1.8 (2)
O1—C2—C3—C4	−6.4 (5)		

Symmetry code: (i) −*x*+1, *y*, −*z*+3/2.

### Hydrogen-bond geometry (Å, º)

**Table:**

*D*—H···*A*	*D*—H	H···*A*	*D*···*A*	*D*—H···*A*
C6—H6*A*···Cl1^ii^	0.96	2.78	3.642 (3)	150

Symmetry code: (ii) −*x*+1/2, −*y*+1/2, −*z*+1.
